# New Cocrystals of Ligustrazine: Enhancing Hygroscopicity and Stability

**DOI:** 10.3390/molecules29102208

**Published:** 2024-05-08

**Authors:** Yifei Xie, Lixiang Gong, Yue Tao, Baoxi Zhang, Li Zhang, Shiying Yang, Dezhi Yang, Yang Lu, Guanhua Du

**Affiliations:** 1 Beijing City Key Laboratory of Drug Target and Screening Research, National Center for Pharmaceutical Screening, Institute of Materia Medica, Chinese Academy of Medical Sciences, Peking Union Medical College, Beijing 100050, China; xieyifei@imm.ac.cn (Y.X.); dugh@imm.ac.cn (G.D.); 2 Beijing City Key Laboratory of Polymorphic Drugs, Center of Pharmaceutical Polymorphs, Institute of Materia Medica, Chinese Academy of Medical Sciences, Peking Union Medical College, Beijing 100050, China; glx15330819543@163.com (L.G.); taoy0215@163.com (Y.T.); zhangbx@imm.ac.cn (B.Z.); zhangl@imm.ac.cn (L.Z.)

**Keywords:** ligustrazine, benzoic acid, cocrystal, stability, hygroscopicity

## Abstract

Ligustrazine (TMP) is the main active ingredient extracted from *Rhizoma Chuanxiong*, which is used in the treatment of cardiovascular and cerebrovascular diseases, with the drawback of being unstable and readily sublimated. Cocrystal technology is an effective method to improve the stability of TMP. Three benzoic acid compounds including P-aminobenzoic acid (PABA), 3-Aminobenzoic acid (MABA), and 3,5-Dinitrobenzoic acid (DNBA) were chosen for co-crystallization with TMP. Three novel cocrystals were obtained, including TMP-PABA (1:2), TMP-MABA (1.5:1), and TMP-DNBA (0.5:1). Hygroscopicity was characterized by the dynamic vapor sorption (DVS) method. Three cocrystals significantly improved the hygroscopicity stability, and the mass change in TMP decreased from 25% to 1.64% (TMP-PABA), 0.12% (TMP-MABA), and 0.03% (TMP-DNBA) at 90% relative humidity. The melting points of the three cocrystals were all higher than TMP, among which the TMP-DNBA cocrystal had the highest melting point and showed the best stability in reducing hygroscopicity. Crystal structure analysis shows that the mesh-like structure formed by the O-H⋯N hydrogen bond in the TMP-DNBA cocrystal was the reason for improving the stability of TMP.

## 1. Introduction

The pharmaceutical cocrystal is defined as a single-phase crystalline solid composed of an active pharmaceutical ingredient (API) and coformers (CCFs) combined in the same crystal lattice through noncovalent interactions in a certain stoichiometric ratio [1,2,3,4]. The formation of cocrystals could improve the poor physicochemical properties of API without modifying the chemical structure [5,6,7,8,9,10,11,12,13,14,15,16].

Ligustrazine (Tetramethylpyrazine, TMP) is the active ingredient in traditional Chinese medicine *Rhizoma Chuanxiong*. used in the treatment of cardiovascular and cerebrovascular diseases [17,18,19]. It is reported that TMP has multiple pharmacological effects, including epilepsy [20], liver fibrosis [21], and obstetrical and gynecological diseases [22]. However, TMP is easily sublimated and hygroscopic in physicochemical properties [23,24,25,26,27].

**Figure 1:**
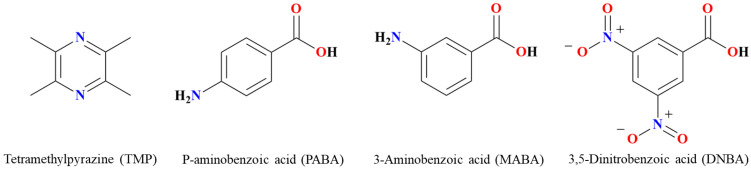
Molecule structures of TMP, PABA, MABA, and DNBA.

The stability of TMP is poor. The N atoms in the structure of TMP can be used as H acceptors to combine the active hydrogen in benzoic acid through the cocrystal transformation method combined with supramolecular and self-assembly principles. Currently, the reported drug cocrystals of TMP include 3,5-dihydroxybenzoic acid [28], 2,6-dihydroxybenzoic acid, 3-nitrophthalic acid, phthalic acid, 3-hydroxybenzoic acid [29], 4-hydroxybenzoic acid [30], etc. It suggests that TMP easily forms cocrystals with benzoic acid compounds, but there is no specific analysis of how benzoic acid compounds with different substituents are connected with TMP to form cocrystals, and the stability of the cocrystals formed by different substituents is compared. If all N atoms in the structure of TMP are connected with CCF to form an inner chain structure, it will contribute to the stability of the cocrystal structure and further improve the stability of TMP. Therefore, we choose p-aminobenzoic acid (PABA) with anti-inflammatory and antibacterial activity as the para-substituted benzoic acid, 3-aminobenzoic acid (MABA) as metasubstituted benzoic acid, and 3,5-dinitrobenzoic acid (DNBA) with antibacterial activity as two meta-substituted benzoic acid compounds, respectively (Figure 1). The characteristics and stability effects of cocrystal formation are systematically discussed.

## 2. Results

### 2.1. Crystal Structure Analysis

Crystals of three cocrystals suitable for single-crystal X-ray diffraction (SXRD) determination were obtained from the solution using the slow evaporation method, namely the TMP-PABA cocrystal (1:2), the TMP-MABA cocrystal (1.5:1) and the TMP-DNBA cocrystal (0.5:1). The crystallographic data of three cocrystals are summarized in Table 1. The CIF for each refinement and checkcif file have been deposited in the Cambridge Structural Database. The crystal structure was elucidated using SXRD.

#### 2.1.1. TMP-PABA Cocrystal (1:2)

**Figure 2:**
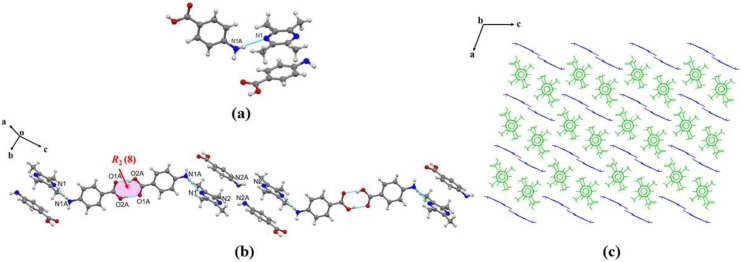
(**a**) Hydrogen bonding interactions in an asymmetric unit; (**b**) weak hydrogen bonds and π-π interactions; (**c**) hydrogen-bonded 3D ladder-like structure.

The obtained data revealed that the TMP-PABA cocrystal (1:2) crystallizes in the monoclinic space group C2/c with one molecule TMP and two molecules PABA in the asymmetric unit, which are formed by hydrogen bonding N1A-H1AA⋯N1 (2.250 Å) and π-π stacking involving the amino groups of the PABA as donors and the nitrogen atoms of the TMP molecule as acceptors as shown in Figure 2a. Two asymmetric units are connected with $R_{2}^{2}$(8) through hydrogen bonds O2AH2A⋯O1A (1.797 Å) to form a hexamer, and then in the projection diagram of the molecule along the a-axis, the neighboring hexamers are linked to each other through hydrogen bonds between N2A and N2 (N2AH2AA⋯N2, 2.437 Å) to form a zigzag chain structure along the c-axis (Figure 2b), the chain structure forms a layered structure along the b-axis (Figure 2c).

#### 2.1.2. TMP-MABA Cocrystal (1.5:1)

**Figure 3:**
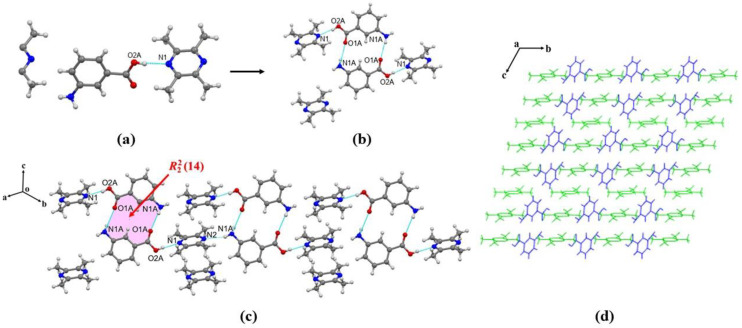
(**a**) Hydrogen bonding interactions in an asymmetric unit; (**b**) pentamer structure; (**c**) hydrogen bonds; (**d**) hydrogen-bonded 3D ladder-like structure.

The single-crystal structure of the TMP-MABA cocrystal crystallizes in a triclinic crystal system and *P*$\overset{-}{1}$ space group. As shown in Figure 3a, an asymmetric unit of the TMP-MABA cocrystal is composed of one and a half molecules of TMP and one molecule of MABA. It can be also viewed that three TMP and two MABA form a pentamer through O2AH2A⋯N1 (1.909 Å) hydrogen bonds between the TMP and the MABA molecules, and two MABA molecules formed $R_{2}^{2}$(14) bond through N1AH1AA⋯O1A (2.193 Å) hydrogen bonds (Figure 3b). Pentamers are connected through hydrogen bonds N1AH1AB⋯N2 (2.349 Å), extending to form a chain-like structure along the b-axis (Figure 3c), the chain structure forms a sandwich layered arrangement along the c-axis (Figure 3d).

#### 2.1.3. TMP-DNBA Cocrystal (0.5:1)

**Figure 4:**
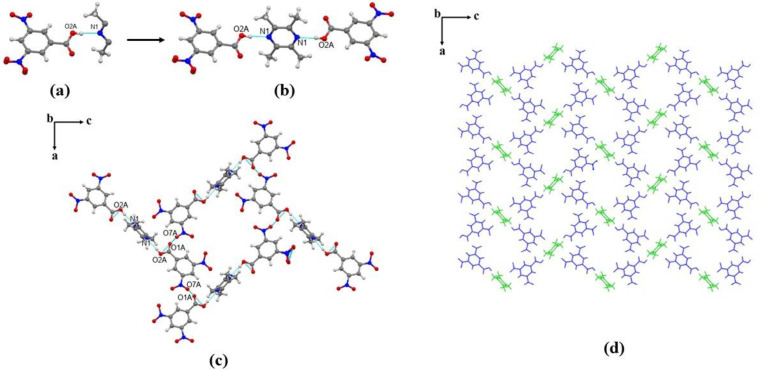
(**a**) Hydrogen-bonding interactions in an asymmetric unit; (**b**) weak hydrogen bonds; (**c**) hydrogen-bonded mesh-like structure (**d**) hydrogen-bonded 3D ladder-like structure (green: TMP, blue: DNBA).

As for the TMP-DNBA cocrystal, it was crystallized in the orthorhombic crystal system and *P*bca space group. Half a TMP molecule and one DNBA molecule constitute an asymmetric unit (Figure 4a). The nitrogen atoms of the amino group at both ends of a TMP molecule are connected to -OH of the carboxyl groups of two DNBA molecules, forming a trimer through hydrogen bonds O2AH2A⋯N1 (1.818 Å) (Figure 4b). Four trimers are connected to form a parallelogram “layer” structure through π-π conjugation (C=O ⋯ O-N+) (Figure 4c). In the projection diagram of molecules along the b-axis, the “layer” structure extends to form a “mesh-like” 3D structure along the a-axis and c-axis directions (Figure 4d).

### 2.2. Hirshfeld Surface Analysis 

**Figure 5:**
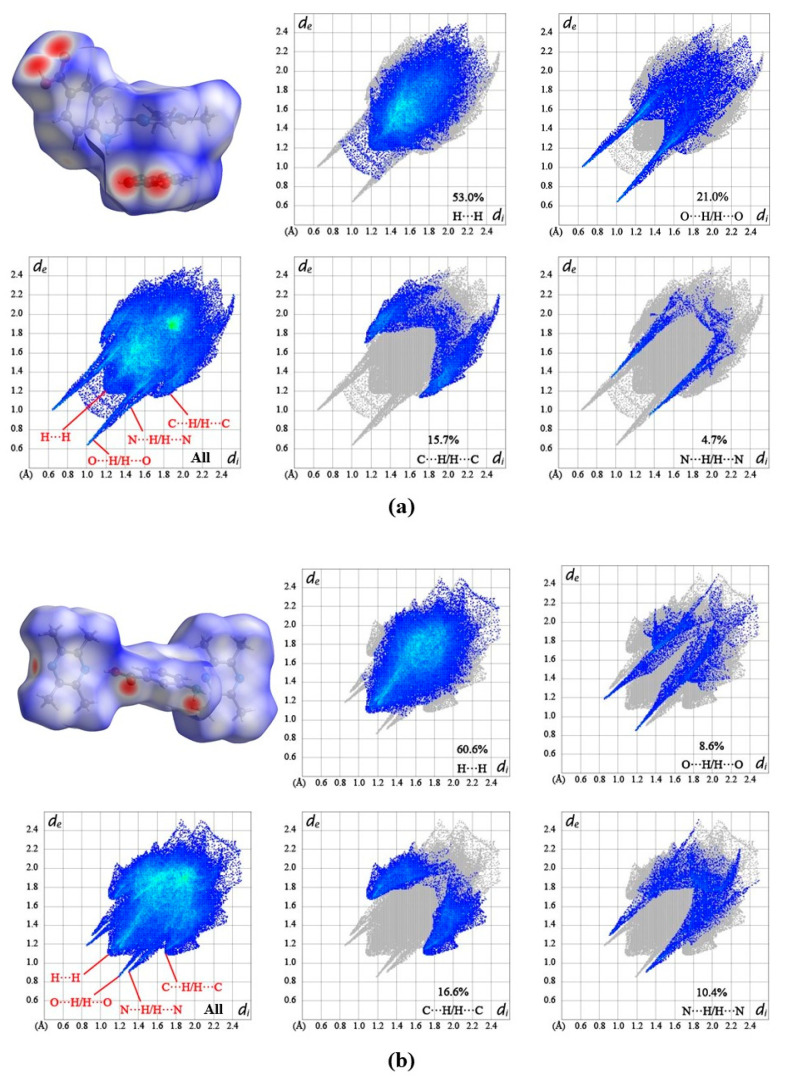

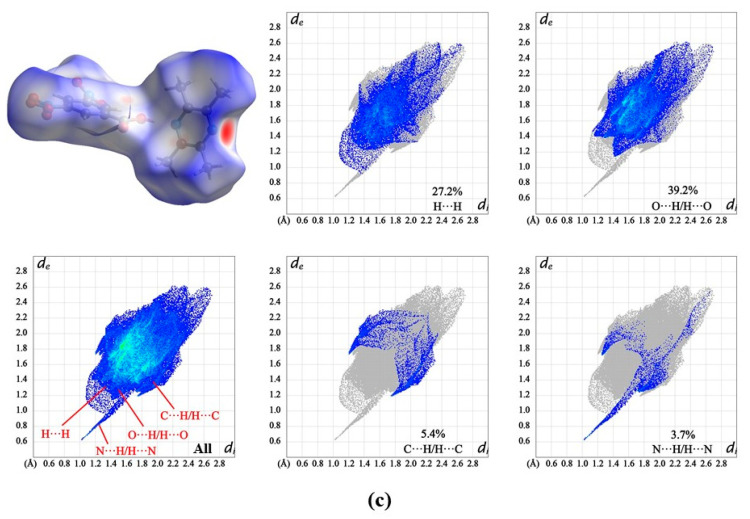
Hirshfeld surface and 2D fingerprint plots of three cocrystals (**a**) TMP-PABA cocrystal. (**b**) TMP-MABA cocrystal. (**c**) TMP-DNBA cocrystal.

Hirshfeld surface was presented under 2D fingerprint plots, which were generated for visualizing the contribution of intermolecular interaction in TMP-PABA cocrystal (a), TMP-MABA cocrystal (b), and TMP-DNBA cocrystal (c) in Figure 5. The strength of the intermolecular force is directly influenced by the color shade, with the red spot signifying the hydrogen bond between the molecules. An intermolecular force that is strong is indicated by a dark color. It can be seen that TMP can only serve as a hydrogen bond acceptor in all 2D fingerprint plots of the cocrystals, while PABA, MABA, and DNBA serve as both hydrogen bond donors and acceptors. As Figure 5 shows, the maximum contributions to the Hirshfeld surface are from H⋯H contacts (53.0% and 60.6%, respectively in TMP-PABA and TMP–MABA). Due to the presence of carboxyl groups in PABA, the hydrogen bond O⋯H/ H⋯O also plays a significant role in intermolecular interactions (21.0%), appearing as two slender sharp peaks at the edge of the fingerprint plots. Based on the finger plots of TMP-PABA cocrystal, the contacts of C⋯H/ H⋯C and N⋯H/ H⋯N accounted for 15.8% and 4.7%, respectively. For TMP–MABA cocrystal, in addition to H⋯H contact, there still exists four interactions including C⋯H/ H⋯C (16.6%), N⋯H/ H⋯N (10.4%), O⋯H/ H⋯O (8.7%). The contributions to the interactions in TMP-DNBA cocrystal are O⋯H/ H⋯O (39.2%), H⋯H (27.2%), C⋯H/ H⋯C (5.4%) and N⋯H/ H⋯N (3.6%). The fingerprint plots of TMP-DNBA cocrystal show that O⋯H/ H⋯O hydrogen bonds are the major contributor to the interactions, which are different from TMP-PABA and TMP-MABA cocrystal. Therefore, the stability of the TMP-DNBA cocrystal is better than that of the TMP-PABA cocrystal and TMP-MABA cocrystal. All cocrystal structures maintain their stable arrangement in space through hydrogen bonding and van der Waals forces.

### 2.3. Theoretical Computation Study

**Figure 6:**
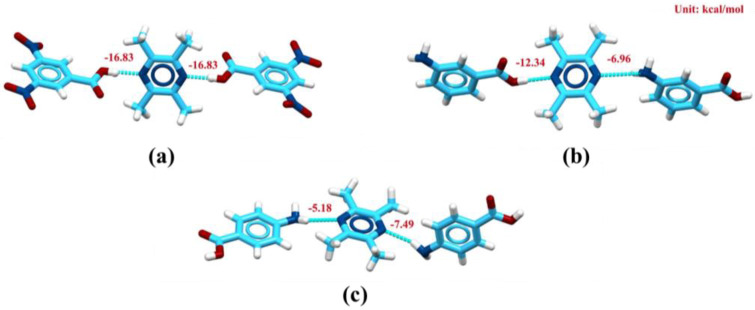
Theoretical calculation between TMP and CCF_S_. (**a**) Interaction between TMP and DNBA. (**b**) Interaction between TMP and MABA. (**c**) Interaction between TMP and PABA.

Theoretical calculation can explain the interaction between API and CCF in cocrystals [31]. By calculating the interaction energy, it is found that when TMP forms a cocrystal with DNBA, the interaction energy between two carboxyl groups and N atoms in TMP is the largest. Interaction energies Eint_1_ and Eint_2_ are −16.83 kcal/mol, respectively, and the total reached −33.66 kcal/mol; When TMP forms cocrystal with MABA, carboxyl and amino in the existing structure react with N in TMP respectively, and the interaction energies Eint_1_ and Eint_2_ are −12.34 kcal/mol and −6.96 kcal/mol respectively, and the total reached to −19.30 kcal/mol. When TMP forms a cocrystal with PABA, amino in PABA reacts with N in TMP. The interaction energies Eint_1_ and Eint_2_ are −5.18 kcal/mol and −7.49 kcal/mol, respectively, and the total reaches −12.67 kcal/mol, which shows that the interaction between carboxyl and N in TMP. The order of interaction strength in the three cocrystals is TMP-DNBA > TMP-MABA > TMP-PABA, which shows that TMP-DNBA is in these three cocrystals. This is also consistent with the results of bond length in cocrystals measured after crystal structure analysis. The bond length of the hydrogen bond connected with TMP in the cocrystal is TMP-DNBA < TMP-MABA < TMP-PABA. Therefore, it shows that when TMP and CCF form a chain structure, it is more beneficial to the stability of the cocrystal (Figure 6).

### 2.4. Powder X-ray Diffraction (PXRD) Analysis

**Figure 7:**
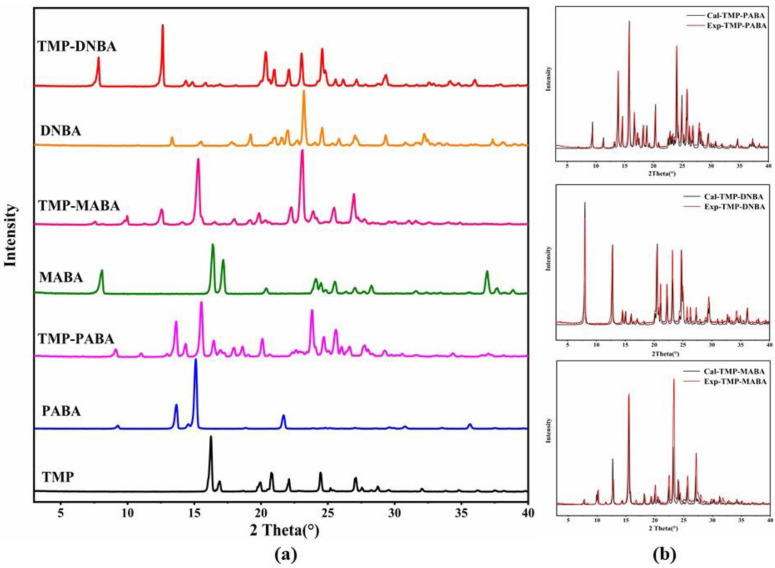
PXRD patterns of the cocrystals and the starting materials of TMP, PABA, MABA, and DNBA. (**a**) Experimental PXRD patterns. (**b**) The experimental and simulated PXRD patterns of three cocrystals.

Each compound produces its unique powder pattern due to its unique crystal structure. Each type of cocrystal of TMP exhibits a unique PXRD pattern that differs from the starting materials of TMP and its three ligands in Figure 7a, suggesting the formation of new crystalline solid phases. TMP showed characteristic diffraction peaks at 2θ values of 16.28°, 16.88°, 19.96°, 20.76°, 22.10°, 24.44°, and 27.10°. The characteristic diffraction peaks of PABA are at 2θ values of 9.26°, 13.68°, 15.12°, and 21.66°. The characteristic diffraction peaks of MABA are at 2θ values of 8.16°, 16.50°, 17.24°, 20.50°, 24.20°, 24.58°, 25.58°, 27.10°, 28.36°, 37.02°, and 37.74°. The characteristic diffraction peaks of DNBA are at 2θ values of 13.34°, 15.50°, 17.82°, 19.22°, 21.08°, 21.52°, 22.02°, 23.20°, 24.56°, 25.82°, 27.02°, 29.34°, 32.20°, and 37.34°. In the diffraction patterns of the three cocrystals of TMP, the characteristic diffraction peaks of TMP have all disappeared. For the TMP-PABA cocrystal, the new diffraction peak has been generated at 2θ values of 9.10°, 11.06°, 14.34°, 15.56°, 17.94°, 18.60°, 20.12°, 23.80°, 24.70°, 25.62°, 26.02°, 26.66°, 27.76°, and 29.28°. For the TMP-MABA cocrystal, the new diffraction peak was generated at 2θ values of 9.90°, 12.52°, 15.24°, 23.02°, 23.82°, 25.4°, and 26.92°. For the TMP-DNBA cocrystal, the new diffraction peak was generated at 2θ values of 7.84°, 12.64°, 14.36°, 14.86°, 20.34°, and 23.04°. The experimental PXRD patterns and the simulated PXRD patterns are presented in Figure 7b. The good consistencies between the experimental PXRD spectra and the simulated PXRD spectra calculated from the SXRD data confirm the high purity of the prepared cocrystals.

### 2.5. Differential Scanning Calorimetry (DSC)

**Figure 8:**
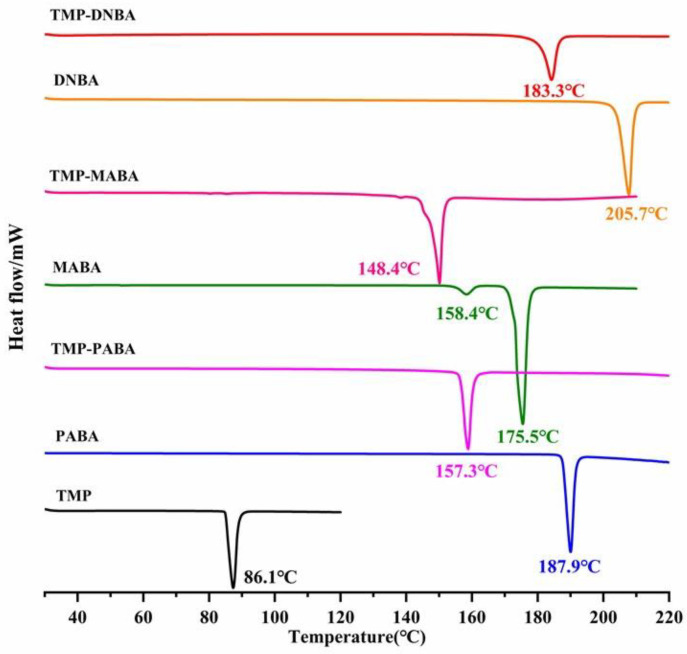
DSC thermograms of the cocrystals and TMP, PABA, MABA, and DNBA.

The thermal behavior of three cocrystals was evaluated through DSC. As shown in Figure 8, three cocrystals were obtained with new narrow endothermic peaks, indicating that the purity of cocrystals obtained by the slurry method is relatively high. Specifically, the DSC thermogram endothermic peaks of TMP-PABA, TMP-MABA, and TMP-DNBA cocrystal are 157.3 °C, 148.4 °C, and 183.3 °C, between the endothermic peaks of API and CCF. Among them, the endothermic peaks of TMP are 86.1 °C and CCF are 187.9 °C (PABA), 175.5 °C (MABA), and 205.7 °C (DNBA), respectively. The emergence of new endothermic peaks proves that there was an interaction between API and CCF, generating new phases. The endothermic peak is related to lattice energy and the stability of the reaction substance [32,33]. The melting points of the three cocrystals are all higher than TMP, which shows that the stability of TMP can be improved by forming cocrystals. The endothermic peak temperature of the TMP-DNBA cocrystal is the highest among the three cocrystals, suggesting the TMP-DNBA cocrystal is the most stable one.

### 2.6. FT-IR Analysis

**Figure 9:**
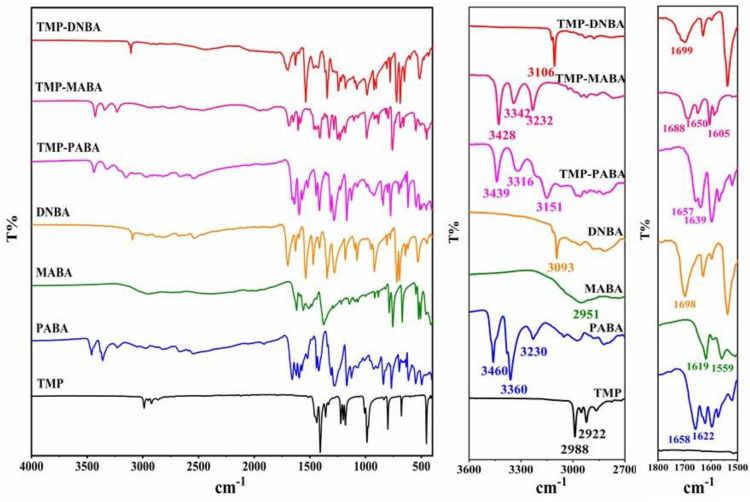
The IR spectra of the TMP cocrystals together with the API and CCFs.

FT-IR is widely used to identify various polymorphic solids due to its sensitivity to subtle changes in crystal structure. The infrared spectra of all samples in this study were characterized by FT-IR to determine the generation of new substances. The IR spectra of the TMP cocrystals together with API and CCF are shown in Figure 9. The characteristic peaks of the imide -C=N group of TMP appear at 2988 cm^−1^ and 2922 cm^−1^. PABA has two moderate absorption peaks of primary amine stretching vibration at 3460 cm^−1^ and 3360 cm^−1^, and primary amine bending vibration at 1658 cm^−1^. The characteristic peaks of the TMP-PABA cocrystal are 3439 cm^−1^, 3316 cm^−1^, and 1657 cm^−1^, which indicates the redshift. It is suggested that a cocrystal is formed by a hydrogen bond between the nitrogen atom of the TMP molecule and the primary amine group of the PABA molecule. During the formation of the TMP-MABA cocrystal, in addition to the hydrogen bonds between the nitrogen atoms on TMP molecules and the hydroxyl groups on the carboxyl groups of MABA molecules, the interaction between the amino and carboxyl groups of MABA molecules formed intermolecular hydrogen bonds, which resulted that the new peaks emerged at 3428 cm^−1^, 3342 cm^−1^, 3232 cm^−1^, and the C=O stretching vibrations and -NH_2_ bending vibrations blue shifted from 1619 cm^−1^ and 1559 cm^−1^ to 1688 cm^−1^ and 1650 cm^−1^. The carboxylic acid group of DNBA shows the C=O stretch at 1678 cm^−1^ and the -OH stretch at 3093 cm^−1^. It suggests that the hydrogen bonds are formed between the nitrogen atoms on TMP molecules and the hydroxyl groups on the carboxyl groups on DNBA molecules in the single crystal structure of the TMP-DNBA cocrystal. Thus, after the formation of the TMP-DNBA cocrystal, blue shifts in amide stretching frequencies and carbonyl frequencies are observed in infrared spectra. These changes in spectral peaks reflect that the imide group of TMP and the carboxyl and amino groups of the CCFs are involved in hydrogen bonds in co-crystallization.

### 2.7. Stability Test

**Figure 10:**
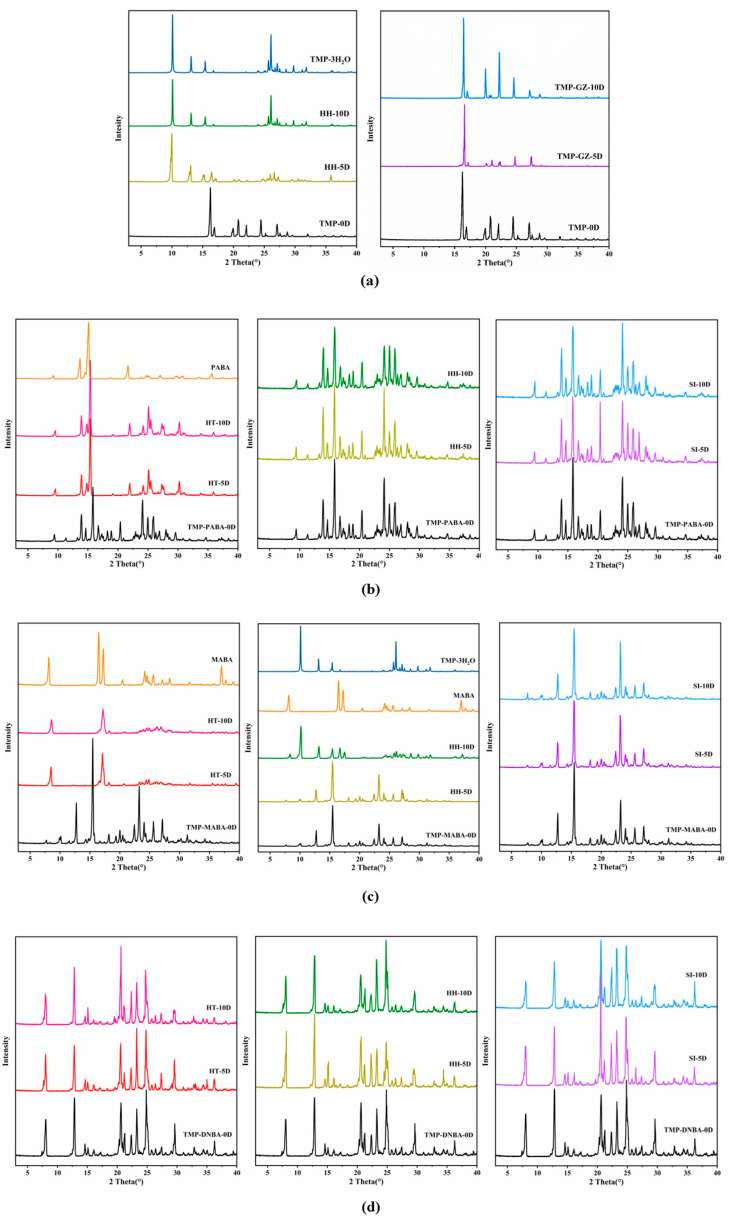
Results of stability test of TMP (**a**) and the cocrystals of TMP-PABA (**b**), TMP-MABA (**c**), and TMP-DNBA (**d**).

TMP was unstable under high temperature (HT, 60 ± 1 °C), high humidity (HH, 95% ± 5%), and strong illumination (SI, 4500 lx ± 500 lx) conditions, as shown in Figure 10a. Specifically, TMP completely evaporated under HT condition for 5 days, due to its melting point. TMP became TMP trihydrate (TMP-3H_2_O) after 5 days of HH conditions. The results of the stability test of cocrystals are shown in Figure 10b–d. The results show that both TMP-PABA cocrystal and TMP-MABA cocrystal are unstable under HT conditions. They will be converted to PABA and MABA, respectively. TMP-MABA cocrystal will be converted to a mixture of MABA and TMP trihydrate under HH conditions. However, the TMP-PABA cocrystal has good stability under HH and SI conditions. TMP-MABA cocrystal can maintain good stability under SI conditions. The TMP-DNBA cocrystal showed the best stability under the three conditions. Overall, the stability of TMP can be significantly improved by generating cocrystals with benzoic acid compounds.

### 2.8. DVS Analysis

**Figure 11:**
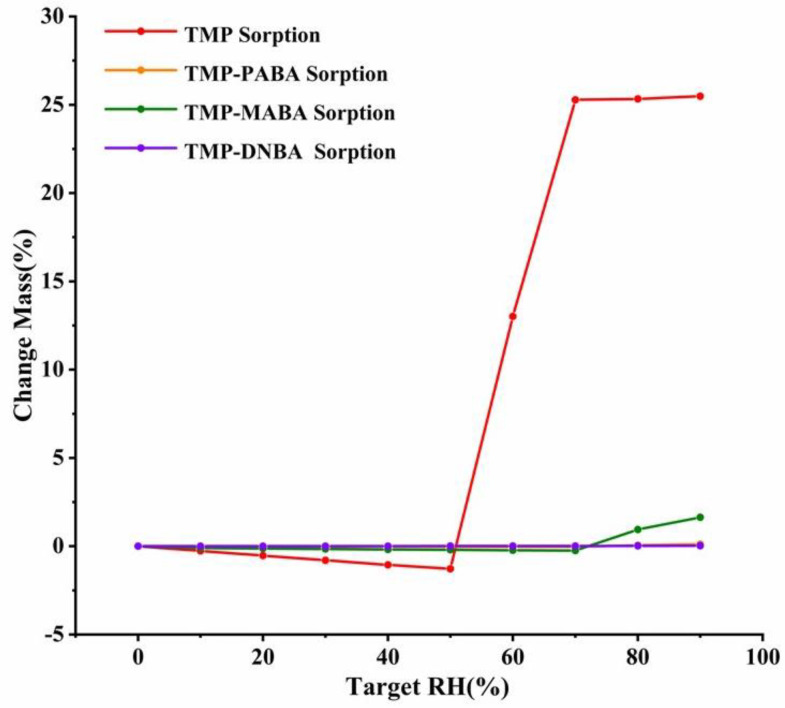
Dynamic vapor sorption isotherms of TMP and cocrystal systems at 25 °C.

DVS can effectively evaluate the hygroscopic stability of drug cocrystals [4,34,35]. Within the RH range of 0~50%, the weight of TMP slightly decreased due to its tendency to sublimate and the condition of nitrogen blowing. Dynamic vapor sorption isotherms of TMP and cocrystal systems at 25 °C are shown in Figure 11. The weight of TMP was reduced by 1.28% at 50% RH. Then, TMP began to absorb water rapidly. When the RH reached 60%, the mass change of TMP was 13.01%. Along with increasing RH up to 70% RH, the weight of the TMP gradually increased to 25.28%. At the range of RH 70~90%, the mass change was in a relatively stable state. At 90% relative humidity, the moisture content of TMP was as high as 25.48%, which indicated that TMP had strong hygroscopicity. However, the moisture content of the TMP-MABA cocrystal was low at 90% relative humidity, which was only 1.64%. The water content of TMP-PABA and TMP-DNBA cocrystals were low at 90% relative humidity, which were 0.12% and 0.03%, respectively. It demonstrated that the hygroscopicity of TMP was greatly improved by forming cocrystals with benzoic acid compounds.

### 2.9. Solubility Experiment

**Figure 12:**
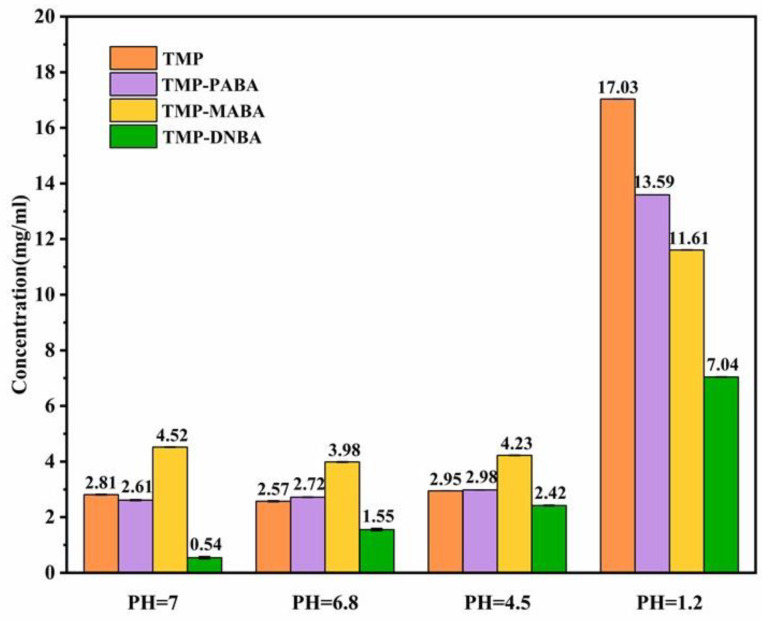
Solubility of TMP, the TMP-PABA cocrystal, the TMP-MABA cocrystal, the TMP-DNBA cocrystal in purified water (pH 7.0), hydrochloric acid solution (pH 1.2), acetate buffer solution (pH 4.5), and phosphate buffer saline (pH 6.8). (*n* = 3).

As shown in Figure 12, it was observed that TMP and three cocrystals had better solubility under pH 1.2 conditions. Acidic media promote the dissociation of TMP, resulting in better solubility in acidic media. The solubility of TMP-PABA cocrystal in pH 7.0, 6.8, and 4.5 buffer was similar to that of TMP. TMP-MABA cocrystal was 1.6 times more soluble than TMP in pH 7.0 buffer, 1.5 times more soluble than TMP in pH 6.8 buffer, and 1.4 times more soluble than TMP in pH 4.5 buffer. All TMP cocrystals were less soluble than TMP in the buffer at pH 1.2. TMP-PABA and TMP-MABA cocrystals were multilayered in their lattices, whereas TMP-DNBA cocrystal was assembled into a mesh-like network structure in the lattice. Therefore, TMP-DNBA is more difficult to detach from the lattice during dissolution than TMP-PABA and TMP-MABA cocrystals, resulting in its lower solubility.

## 3. Experimental Section

### 3.1. Materials

Both TMP (purity > 98%) and DNBA (purity > 98%) were purchased from Hubei Wande Chemical Co., Ltd. (Tianmen, China). PABA (purity > 99%) was purchased from Wuhan Yuancheng Technology Development Co., Ltd. (Wuhan, China). MABA acid (purity > 99%) was purchased from InnoChem Science & Technology Co., Ltd. (Beijing, China). All analytical grade solvents were purchased from Beijing Chemical Reagent Factory (Beijing, China). The methanol used in high-performance liquid chromatography analysis was chromatographically pure and was purchased from Honeywell Burdick & Jackson Co. (Ulsan, Korea) The solid reagents, including potassium dihydrogen phosphate, sodium hydroxide, and anhydrous sodium acetate, used for the preparation of buffer solution for solubility research, were all analytical pure and were purchased from Beijing Chemical Reagent Factory (Beijing, China).

### 3.2. Preparations

#### 3.2.1. Preparation of TMP-PABA Cocrystal (1:2)

The powder sample of TMP-PABA cocrystal (1:2) was prepared by the slurry method. TMP (817.2 mg, 6 mmol), PABA (1645.7 mg, 12 mmol), and 8 mL acetonitrile were added in a glass vial, with a stirring rate of 300 rpm for 2 days at room temperature. The light yellow powder sample was obtained after drying at room temperature. The single crystal of the TMP-PABA cocrystal (1:2) was obtained by the slow evaporation method. TMP (13.6 mg, 0.1 mmol) and PABA (27.4 mg, 0.2 mmol) were dissolved in 10 mL acetone. Pale yellow transparent block-shaped crystals were obtained at 23 °C for 7 days.

#### 3.2.2. Preparation of TMP-MABA Cocrystal (1.5:1)

The powder sample of TMP-MABA cocrystal (1.5:1) was prepared by the slurry method. TMP (1634.3 mg, 12 mmol), MABA (1097.2 mg, 8 mmol), and 8 mL acetonitrile were added in a glass vial, with a stirring rate of 300 rpm for 2 days at room temperature. The light pink powder was obtained after drying at room temperature. The single crystal of the TMP-MABA cocrystal (1.5:1) was obtained by the slow evaporation method. TMP (20.4 mg, 0.15 mmol) and MABA (13.7 mg, 0.1 mmol) were dissolved in 2 mL ethyl acetate. Colorless transparent block crystals were obtained at 23 °C for 5 days.

#### 3.2.3. Preparation of TMP-DNBA Cocrystal (0.5:1)

The powder sample of TMP-DNBA cocrystal (0.5:1) was prepared by the slurry method. TMP (544.8 mg, 4 mmol), DNBA (1697.0 mg, 8 mmol), and 8 mL acetonitrile were added in a glass vial, with a stirring rate of 300 rpm for 2 days at room temperature. The white powder sample was obtained after drying at room temperature. The single crystal of the TMP-PABA cocrystal (1:2) was obtained by the slow evaporation method. TMP (13.6 mg, 0.1 mmol) and DNBA (42.2 mg, 0.2 mmol) were dissolved in 2 mL methanol. Colorless transparent columnar-shaped crystals were obtained at 23 °C for 7 days.

### 3.3. Single-Crystal X-ray Diffraction (SXRD)

SXRD experiments were performed at ambient temperature on a Rigaku Micromax 002+ CCD diffractometer (Rigaku, The Woodlands, TX, USA) and a graphite monochromator using Cu Kα radiation (λ = 1.54184Å). All crystal structures were solved by Olex2 1.5 Software using the direct method and refined by the full-matrix least-squares method on F^2^ with anisotropic displacement parameters [36,37,38]. All the non-hydrogen atoms were refined anisotropically. The H atoms bound to the O atoms are located using Fourier analysis and geometric calculation. The program Mercury (2023.3.0) was applied to generate molecular graphics. The Hirshfeld surface and 2D fingerprint plot analysis of TMP-PABA, TMP-MABA, and TMP-DNBA were analyzed by Crystal Explorer 17.5 Software.

### 3.4. Theoretical Computation Study

The geometry optimization of hydrogen atoms and single-point energy calculations were conducted using the Gaussian package at the B3LYP-D3BJ/6-311G (d, p) and M06-2X(D3)/def2-TZVP levels. Concurrently, the interaction energies between TMP and other molecules within the respective cocrystal were calculated at the same computational level, employing the counterpoise correction method.

### 3.5. Powder X-ray Diffraction (PXRD)

PXRD experiments were performed using a Rigaku D/MAX-2550 diffractometer (Rigaku, Tokyo, Japan) with a graphite monochromator using Cu Kα radiation (λ = 1.54184Å). The tube voltage and amperage were set to 40 kV and 150 mA, respectively. Diffraction patterns were collected in a continuous scanning mode in the 2θ range 3–40° using a step size of 0.02° (2θ) and a scanning speed of 8°/min. The data were processed using JADE 6.0 software. [39,40] The simulated PXRD pattern was obtained using the Mercury (2023.3.0) software. Chemical and structural consistency between bulk sample powders and single crystals was verified by comparing the experimental and simulated PXRD patterns.

### 3.6. Differential Scanning Calorimetry (DSC) 

The DSC spectra were recorded using a 1/500 differential scanning calorimeter (Mettler Toledo, Greifensee, Switzerland), and STARe Evaluation 13.0 software Powder samples were placed in aluminum crucibles and heated at 10 K/min and the range of programmed temperature was 30 °C to 220 °C. 

### 3.7. Fourier Transform Infrared (FT-IR) Spectroscopy 

All FT-IR measurements were performed using a PerkinElmer Spectrum 400 Fourier transform infrared spectrophotometer (PerkinElmer, Waltham, MA, USA) and attenuated total reflection sampling attachment. The infrared spectrum was recorded in a scanning range of 400 to 4000 cm^−1^ with 16 scans and a resolution of 4 cm^−1^ and processed using the PerkinElmer software kit (6.3.5 v.).

### 3.8. Stability Experiment

PXRD evaluated the stability of the TMP-PABA cocrystal (1:2), TMP-MABA cocrystal (1.5:1), and TMP-DNBA cocrystal (0.5:1) under high temperature (60 ± 1 °C), high humidity (95% ± 5%), and strong illumination (4500 lx ± 500 lx) conditions. 

### 3.9. Dynamic Water Vapor Sorption (DVS) 

Moisture sorption isotherms were collected on a Surface Measurement Systems DVS Resolution (SMS, London, UK) at 25 ± 0.1 °C. DVS technology calculates the equilibrium amount of vapor sorption and desorption by measuring the change in sample mass when the sample reaches the equilibrium state under conditions of a certain temperature and different relative humidity. Before data collection, each sample was dried under nitrogen, purged until a constant weight was attained, which was assumed to be the dry mass of the sample. The sample equilibrated at each step with equilibration criteria of either dm/dt ≤ 0.002% or a maximum equilibration time of 300 min. Then, the relative humidity (RH) varied in the range of 0–90–0% with a step size of 10%. When one of the criteria was met, the relative humidity (RH) was changed to the next stage. Finally, it drew the sorption–desorption isotherm according to their relationship to study the trend and extent of water vapor sorption on samples.

### 3.10. Solubility Experiments

Solubility tests were carried out by adding an excess TMP, TMP-PABA, TMP-MABA, and TMP-DNBA under pH 1.2, 4.5, 6.8, and 7.0 buffer solutions. The suspensions were kept for continuously shaking in vials containing 2 mL of experimental media at 37 ± 0.5 °C for 48 h, then stewing for 24 h. The supernatant was diluted by 30 times and then filtered using 0.22 μm nylon filters. TMP, TMP-PABA, TMP-MABA, and TMP-DNBA of the resulting filtrates were assayed by high-performance liquid chromatography (HPLC). 

### 3.11. HPLC Method

TMP and cocrystals were quantified using a quaternary pump Agilent HPLC system (1260 series) equipped with a UV detector (Agilent Technologies, Santa Clara, CA, USA). A KromasilCl8 (4.6 mm × 250 mm, 5 um) column was used for chromatographic separation. The mobile phase was pure water (pH 7.0) and methanol in 40:60 ratios. Analysis was performed at a flow rate of 1 mL/min and a detector wavelength of 280 nm. The injection volume was 10 μL. 

## 4. Conclusions

The purpose of this work was to investigate the co-crystallization of benzoic acid compounds with TMP to enhance the stability and improve the physicochemical qualities. In this study, cocrystals of TMP with three benzoic acid compounds were prepared by the slurry method, and three new cocrystals were obtained by the slow solvent evaporation method. SXRD, PXRD, IR spectroscopy, and DSC were used to characterize the cocrystals to determine three novel structures. TMP-PABA cocrystal forms N-H⋯N and O-H⋯O hydrogen bonds through nitrogen atoms on the tertiary amine of TMP and the carboxyl group of PABA. The molecules in the TMP-MABA cocrystal and TMP-DNBA cocrystal are connected by O-H⋯N hydrogen bond between the tertiary amine group of TMP and hydroxyl groups on carboxyl of PABA and DNBA. DSC, stability test and DVS results show that cocrystal exhibits better hygroscopicity and thermal stability than TMP. The TMP-DNBA cocrystal shows the best stability among these cocrystals and is also supported by the equilibrium solubility result. Therefore, for the solid drugs of TMP, selecting benzoic acid compounds to form cocrystals with a mesh structure via an O-H⋯N hydrogen bond is a method to improve the stability and related oral dosage form of TMP. The theoretical calculation shows that TMP and DNBA have the largest intermolecular interaction energy, which also shows that TMP-DNBA has the strongest cocrystal stability.

## Figures and Tables

**Table 1:** Crystallographic parameters of TMP cocrystals.

	TMP-PABA	TMP-MABA	TMP-DNBA
empirical formula	C_8_H_12_N_2_·2(C_7_H_7_NO_2_)	1.5(C_8_H_12_N_2_)·C_7_H_7_NO_2_	0.5(C_8_H_12_N_2_)·C_7_H_4_N_2_O_6_
molecule weight	410.47	341.43	280.22
crystal size (mm)	0.21 × 0.23 × 0.25	0.20 × 0.20 × 0.20	0.10 × 0.10 × 0.30
temperature (K)	302	304	293
description	block	block	columnar
crystal system	monoclinic	triclinic	orthorhombic
space group	*C*2/c	*P* $\overset{-}{1}$	*P*bca
a (Å)	23.217 (1)	9.017 (1)	13.888 (1)
b (Å)	7.388 (1)	9.449 (1)	8.550 (1)
c (Å)	25.917 (1)	12.144 (1)	22.094 (1)
α (deg)	90	105.438 (2)	90
β (deg)	100.597 (2)	97.994 (2)	90
γ (deg)	90	98.473 (2)	90
volume (Å^3^)	4369.66 (17)	968.98 (4)	2623.46 (8)
Z	8	2	8
density (g/cm^3^)	1.248	1.170	1.419
independent reflections	4445	3906	2659
reflections with I > 2σ(I)	3485	3158	2376
R_int_	0.045	0.040	0.036
final R, wR (F^2^) value [I > 2 σ(I)]	0.059, 0.185	0.055, 0.176	0.044, 0.130
goodness-of-fit on F^2^	1.046	1.064	1.037
CCDC numbers	2,339,277	2,339,276	2,339,275

The deviation value is represented in brackets.

## Data Availability

The data on the compounds are available from the authors.
